# Effect of mindfulness-based interventions on anxiety, depression, and stress in patients with coronary artery disease: a systematic review and meta-analysis of randomized controlled trials

**DOI:** 10.3389/fpsyg.2024.1435243

**Published:** 2024-07-31

**Authors:** Hanani Abdul Manan, Imtiyaz Ali Mir, Syeda Humayra, Rong Yuen Tee, Deepak Thazhakkattu Vasu

**Affiliations:** ^1^Department of Radiology, Functional Image Processing Laboratory, Universiti Kebangsaan Malaysia, Kuala Lumpur, Malaysia; ^2^Department of Physiotherapy, M. Kandiah Faculty of Medicine and Health Sciences, Universiti Tunku Abdul Rahman, Kajang, Selangor, Malaysia

**Keywords:** anxiety, coronary artery disease, depression, mindfulness-based stress reduction, stress

## Abstract

**Background:**

Adopting lifestyle interventions is pivotal in coronary artery disease (CAD) management and prevention to amplify cardiovascular and mental well-being. This study aims to quantify the effect of mindfulness-based interventions (MBIs) on anxiety, depression and stress in CAD patients.

**Methods:**

A systematic review and meta-analysis of randomized controlled trials (RCTs) was conducted by searching four electronic databases (PubMed, CENTRAL, Scopus, and Science Direct) through December 2023. The risk of bias was assessed using the PEDro tool, and the study outcomes were expressed as standard mean difference at 95% CI.

**Results:**

Out of 1838 yielded results, eight RCTs involving 623 participants with a mean age of 56.96 ± 4.89 met the prespecified eligibility criteria. The pooled results showed a statistically significant and beneficial effect of MBIs on CAD patients’ mental health status in regards to anxiety (SMD = −0.83; 95% CI [−1.19, −0.46], *p* < 0.001), depression (SMD = − 0.86; 95% CI [−1.14, −0.58], *p* < 0.001), and stress (SMD = −0.69; 95% CI [−1.27, −0.12], *p* = 0.02). The subgroup sensitivity analyses based on the region (Asia vs. Europe) indicated a statistically non-significant subgroup effect of MBIs on anxiety (*I*^2^ = 63.9%, *p* = 0.10) and depression (*I*^2^ = 25.8%, *p* = 0.25), and a significant effect on stress (*I*^2^ = 80.0%, *p* = 0.03). Although the methodological quality of the trials was generally satisfactory, all studies lacked allocation concealment and blinding. Additionally, gender imbalances, and inadequate follow-up may have potentially compromised the validity of the trials.

**Conclusion:**

Mindfulness-based interventions are beneficial for improving CAD patients’ anxiety, depression and stress symptoms. Nevertheless, it is imperative to conduct more rigorous and robust studies with an equal gender ratio and long-term follow-up.

## 1 Introduction

Coronary artery disease (CAD), also referred to as coronary heart disease (CHD), is a cardiovascular disorder (CVD) in which the coronary arteries supplying oxygenated blood to the cardiac muscles, suffer from blockage and narrowing due to the build-up of atherosclerotic plaque. This impairs coronary circulation and can result in myocardial infarction (MI) (Shahjehan and Bhutta, 2024). Worldwide, CAD is the primary cause of mortality and Disability Adjusted Life Years (DALYs) (Ralapanawa and Sivakanesan, 2021). According to the British Heart Foundation (2024), approximately 110 million men and 80 million women around the world are affected by CAD and accounts for one in six deaths globally (British Heart Foundation, 2024). This condition claimed the lives of an estimated 9 million people annually, making it the leading cause of death globally in 2019 (WHO, 2024). Another significant contributor owing up to the worldwide burden of disease is mental illness, which accounts for 13% of the total global burden of disease (WHO, 2011). One of the main mental health disorders is depression, which affects about 300 million individuals globally across all age groups, and by 2030, it is predicted to overtake all other causes of disability (WHO, 2011, 2017). A meta-analysis revealed that mental diseases account for almost 8 million fatalities annually, or 14.3% of all global deaths (Walker et al., 2015). Furthermore, there seems to be an interesting bidirectional connection between mental illness and CAD. Increasing evidence indicates that patients with CAD have a higher prevalence of mental illnesses (De Hert et al., 2018; Kang and Malvaso, 2023). Mental health issues such as anxiety, depression and stress are more prevalent among patients with CAD (Cohen et al., 2015). It is reported that poor psychological health increases cardiovascular risk by activating biological pathways through the hyperactivity of the hypothalamus–pituitary–adrenal axis and the sympathetic nervous system (Wirtz and von Känel, 2017; Levine, 2022). In addition, scientific reports have also shown that CAD patients suffering from psychological distress are more likely to experience worsened quality of life (QoL), adverse cardiovascular events, and increased all-cause mortality (Meijer et al., 2011; Komalasari et al., 2019).

Early screening and management of comorbid psychiatric disorders in cardiac patients are equally important since CAD and mental illnesses are accountable for the world’s leading cause of morbidity and mortality (De Hert et al., 2018). Therefore, individuals with cardiovascular risk factors must effectively manage their condition through lifestyle modifications, psychological interventions, appropriate medications, and regular medical check-ups to reduce the chances of developing coronary artery disease and possible mental health issues. Mindfulness-based interventions (MBIs) are proven and evidence-supported programs that integrate mindfulness meditation and body awareness to assist individuals in effectively coping with stress, anxiety, and various psychological as well as physical symptoms (Hofmann and Gómez, 2017; Bhattacharya and Hofmann, 2023; Laura, 2023; Javadzade et al., 2024). Participants in mindfulness programs are directed to enhance their self-awareness about thoughts, emotions, sensations, and the surrounding environment through diverse meditation techniques, including focused attention on the breath, body scan, and loving-kindness meditation. The increased awareness allows individuals to respond to internal/external stressors and challenges more effectively and with greater resilience. Mindfulness-based stress reduction (MBSR) has demonstrated positive effects on various aspects of mental health including mitigation of stress, anxiety, and depressive symptoms (Goldin and Gross, 2010; Chen et al., 2020). It has also facilitated in improving sleep quality, increasing overall well-being, and enhancing cognitive function and focus (Whitfield et al., 2022; Javadzade et al., 2024). A previous systematic review and meta-analysis of MBIs among CVD population deduced the short-term beneficial impacts of MBIs both psychologically and physiologically (Scott-Sheldon et al., 2020).

From the broad array of scientific evidence, MBIs indeed provide beneficial and positive effects in managing mental disorders. However, the majority of the previous research did not solely focus on CAD and targeted different patient populations with diabetes, heart failure, and CVDs in general (Scott-Sheldon et al., 2020; Zou et al., 2020; Marino et al., 2021; Hamasaki, 2023; Zhang et al., 2024). Therefore, to address this research gap and comprehend the efficacy of MBIs toward CAD patients’ mental health status, this systematic review and meta-analysis aims to investigate the effect of MBIs on anxiety, depression, and/or stress among CAD patients.

## 2 Materials and methods

### 2.1 Search strategy and study selection

This study followed the principles of the Preferred Reporting Items for Systematic Reviews and Meta-Analyses (PRISMA) and was registered prospectively on PROSPERO with the registration number CRD42024504938. The Population, Intervention, Comparison, Outcome, and Study Selection (PICOS) criteria were used to determine the eligibility of a study for inclusion in this meta-analysis as shown in Table 1. Adult participants diagnosed with CAD, including those with a history of MI, percutaneous coronary intervention, coronary artery bypass grafting, and individuals with stable or unstable angina were selected. Randomized controlled trials (RCTs) that assessed the effect of MBIs on mental health status (anxiety, depression, and/or stress) were included. Studies with a non-randomized control design, observational or case studies, systematic and non-systematic reviews, meta-analyses and books or book chapter were excluded. Interventions that focused on mindfulness as the main treatment aspect such as programs incorporating the development of mindfulness skills into organized training programs, e.g., MBSR and mindfulness-based art therapy (MBAT) were considered for selection. Adapted, shortened, or amended versions of standard mindfulness programs, such as focused meditation or movement meditation without any particular restrictions on the treatment type, duration of treatment, and dosage of treatment and mode of delivery were included. Articles or records which were not reported in English were excluded due to lack of resources for translation. Studies that only reported the abstracts without full-text publication, or incomplete and irrelevant outcomes were excluded too. Research with duplicated or overlapping data that were common from other sources, and patients with other heart conditions, pulmonary disease or renal impairment were also excluded.

**Table 1:** PICOS framework for the study.

PICOS	Inclusion criteria	Exclusion criteria
Population	Adults diagnosed with coronary artery disease, including those with a history of myocardial infarction, percutaneous coronary intervention, coronary artery bypass grafting, and individuals with stable or unstable angina.	Patients with other heart conditions (e.g., valvular disease, septal defect, cardiomyopathy, arrhythmias etc.), any pulmonary disorder or renal disease.
Intervention	Mindfulness-based interventions focusing on mindfulness-based stress reduction and mindfulness-based art therapy, and adopted versions of standard mindfulness program such as focused meditation and movement meditation.	Interventions that included spiritual meditation, mantra meditation, or transcendental meditation.
Comparison	Active or inactive control group.	Absence of control group.
Outcome	Anxiety, depression, and/or stress.	None.
Study design	Randomized clinical trials (RCTs).	Non-randomized controlled trails, observational studies, case studies, systematic and non-systematic reviews, meta-analyses and books or book chapter.

**Figure 1:**
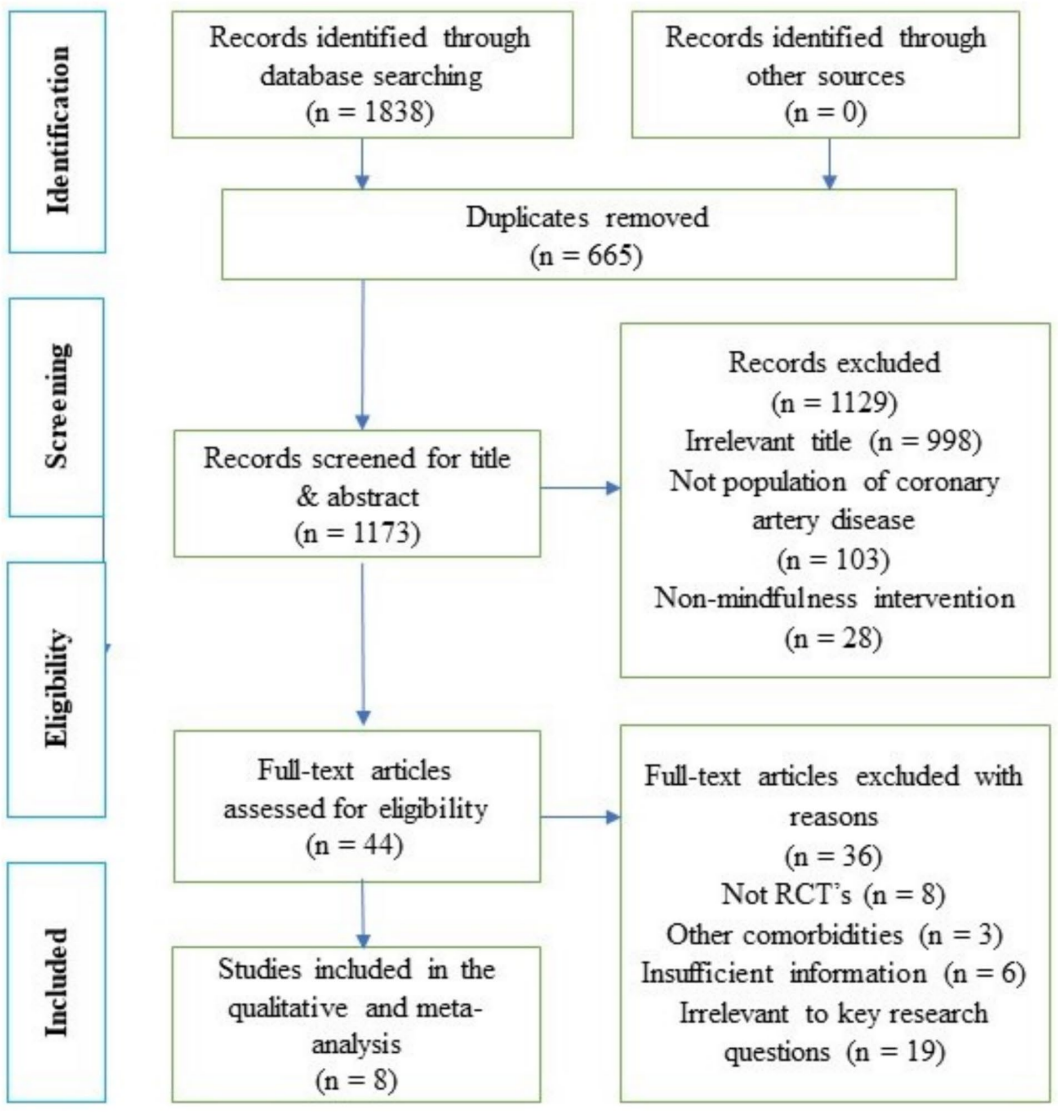
Flow chart of study identification.

Four electronic databases (PubMed, CENTRAL, Scopus, and Science Direct) were used to search potential articles from inception until December 2023. Before initiating the search and screening, relevant keywords, synonyms, and suitable terminologies were finalized. Boolean operator like “OR” was used to incorporate alternate synonyms to broaden the search, while “AND” was used to conjoin important key words. Search keywords included the following: mindfulness, OR mindfulness meditation, OR mindfulness-based stress reduction, mindfulness-based intervention, OR mindfulness-based cognitive therapy, AND mental health, OR emotional well-being, OR psychological well-being, OR psychological health, OR anxiety, OR depression, OR stress AND coronary artery disease, OR coronary heart disease, OR ischemic heart disease, OR myocardial infarction OR percutaneous coronary intervention OR coronary artery bypass surgery. Two independent reviewers were involved in searching the databases and screening the articles against the predefined eligibility criteria. In case of any disagreements between the two reviewers, a third author was involved and a mutual consensus was made to resolve the issues. Figure 1 shows the complete flowchart of the study identification process for this systematic review. Firstly, 1,838 records were initially identified by searching the databases. The articles were then screened for any duplicate results. A total of 665 duplicates were identified and excluded. Finally, 1,173 articles were screened for title and abstracts eligibility. Subsequently, the full-text of these articles were screened to confirm whether they meet the eligibility criteria. In the first stage, 1,129 articles were excluded due to various reasons such as articles having irrelevant titles (*n* = 998); not population of CAD (*n* = 103); and research using non-mindfulness interventions (*n* = 28). Thereafter, 44 articles were screened in the second stage of the process, whereby 36 articles were excluded due to various reasons, e.g., research design was not RCT (*n* = 8); articles contained insufficient information (*n* = 6); recruited participants had other health conditions as highlighted in exclusion criteria (3); articles that were irrelevant to key research questions (*n* = 19). Finally, a total of eight articles were used in the qualitative and meta-analysis.

### 2.2 Data extraction and data synthesis

Two data extraction tables were prepared on an Excel spreadsheet. The first table contained the summary of the general characteristics of studies and the second table contained the intervention characteristics of studies. Data from the articles were extracted and filled in the tables by two reviewers independently. The data tables were then rechecked and validated by a third reviewer. Data synthesis was performed using qualitative synthesis and meta-analysis. Qualitative synthesis was used to analyze and integrate data, and provide a general summary of characteristics and findings of the included studies. Relationship among studies was examined in detail, delving into patterns and exploring the variations between them. The strengths and weaknesses of a study were also identified, and any risk of bias in the included studies was evaluated and expressed utilizing PEDro scale. Meta-analysis was performed using the Review Man 5.4 software developed under Cochrane. Since the outcome variables were continuous and measured in different ways, which in this case, were the various psychometric scale scores utilized in the assessment of mental health (anxiety, depression, and stress), the effect index utilized was a standard mean difference (SMD), and each effect’s point estimates were based on a 95% confidence interval (CI). This meta-analysis employed the random-effects model considering potential heterogeneity within the included studies. The study’s heterogeneity was examined by carrying out the sensitivity analysis, which involved repeating the primary analysis of effect of MBIs on mental health status based on region of study (Asia vs. Europe) and the results were coupled with *I*^2^ statistics. *p* < 0.05 was considered as statistically significant. In addition, the effectiveness of mindfulness intervention in the selected trials was analyzed by determining the effect size—*Z* (Cohen’s *d*). Effect size is considered small when *Z* is more than 0.2 but less than 0.5, effect size is medium when z is more than 0.5 but less than 0.8 and effect size is large when *Z* is above 0.8.

## 3 Results

### 3.1 Study and participant characteristics

Details of study characteristics including first author name with year and country, study design, study comparator, sample size, mean age ± SD (years) in experimental and control groups, ethnicity, participants analyzed in experimental and control groups, and male to female ratio are presented in Table 2. A total of eight studies were included in this review and six trials were conducted in Asia and two in Europe. Out of 623 participants that were analyzed, male participants (*n* = 450) accounted for 72.2%, while female participants (*n* = 173) constituted 27.8%, and one study recruited only male participants (Parswani et al., 2013). Five studies included patients with variating CAD conditions (Michalsen et al., 2005; Parswani et al., 2013; Jang et al., 2018; Nasiri et al., 2020; Wu et al., 2023), and the other three studies had patients who have undergone revascularization surgery (Nyklíček et al., 2014; Hou et al., 2019; Liang et al., 2019). The mean age of the participants ranged from 47 to 65 years old. Three studies presented patients of Asian ethnicity (Jang et al., 2018; Hou et al., 2019; Liang et al., 2019), and one study had Caucasian participants (Nyklíček et al., 2014), while the others did not report the ethnic background. The sample size ranged from 31 to 116 participants, and all studies implemented an RCT with two parallel groups.

**Table 2:** General characteristics of studies.

Author, year and country	Study design	Study comparator	Sample size	Mean age ± SD (years) in experimental & control groups	Ethnicity	Participants analyzed in experimental & control groups	Male/Female
Hou 2019 (China)	RCT with two parallel group	Wait list control group	70	Intervention: 60.40 ± 10.86	Asian	30/31	49/12
				Control: 56.97 ± 10.99			
Jang 2018 (South Korea)	RCT with two parallel group	Waitlist control group	44	Intervention: 64.81 ± 9.19	Asian	23/21	30/14
				Control: 65.3 ± 7.12			
Wu 2023 (China)	RCT with two parallel groups	Early cardiac rehab	100	Intervention: 59.72 ± 6.43	Not reported	50/50	72/28
				Control: 60.42 ± 7.12			
Liang 2019 (China)	RCT with two parallel group	Conventional nursing	116	Intervention: 55.12 ± 6.17	Asian	58/58	65/51
				Control: 55.41 ± 6.25			
Michalsen 2005 (Germany)	RCT with two parallel group	Written advice-only group	105	Intervention: 59.0 ± 8.7	Not reported	48/53	78/23
				Control: 59.8 ± 8.6			
Nasiri 2020 (Iran)	RCT with two parallel group	Routine care	64	Intervention: 52.71 ± 10.94	Not reported	32/32	38/26
				Control: 52.16 ± 13.56			
Nyklíček 2014 (Netherlands)	RCT with two parallel group	Self-help (Booklet provided)	114	Intervention: 55.4 ± 7.3	Caucasian	55/52	88/19
				Control: 56.3 ± 7.3			
Parswani 2013 (India)	RCT with two parallel group	Routine cardiac care	31	Intervention: 47.27	Not reported	15/15	All participants were male
				Control: 50.60			

### 3.2 Intervention characteristics

Intervention characteristics of the selected trials included intervention type with dosage and duration, mode of delivery, post-test outcome differences between experimental and control group, significance level, effect size, follow-up phase and authors’ conclusion as depicted in Table 3. Seven studies adopted MBSR intervention and one study (Jang et al., 2018) adopted MBAT (MBSR + mindfulness art therapy). The study settings were either hospital-based (Michalsen et al., 2005; Liang et al., 2019; Nasiri et al., 2020), or hybrid involving both hospital and home sessions through telephone (Hou et al., 2019). The most common kinds of mindfulness interventions used were body scans, sitting meditation, breathing space, and walking meditation. The intervention dosage prescribed varied largely across the included studies. The overall duration of intervention ranged from 5 to 7 days as the shortest (Liang et al., 2019; Wu et al., 2023) and the longest was held up to a year (Michalsen et al., 2005), and frequency ranged from twice daily to once weekly or once every 2-week with each session lasting for a minimum of 30 min to a maximum of 3 h. The mode of delivery for interventions also differed. Four trials used group sessions and the number of participants in groups ranged from 5 to 12 people (Michalsen et al., 2005; Nyklíček et al., 2014; Jang et al., 2018; Liang et al., 2019). Three studies employed individual mode of delivery through face-to-face sessions (Parswani et al., 2013; Hou et al., 2019; Wu et al., 2023), while one study did not report whether individual or group sessions were held (Nasiri et al., 2020). Regarding the intervention type, duration, and dosage, study by Hou et al. (2019) used MBSR, with a total intervention duration of 6 weeks, biweekly one session of 30–40 min; trial by Jang et al. (2018) employed MBAT for 12 weeks, weekly one session of 45 min; experiment by Wu et al. (2023) utilized MBSR for 5–7 days, two sessions/day, each session for 30 min; research by Liang et al. (2019) used MBSR, with a total intervention time of 7 days, daily one session for 3 h; trial by Michalsen et al. (2005) deployed MBSR, intervention duration lasted 1 year with 3 h weekly of session for the first 10 weeks, then 2 h of weekly session for the next 10 weeks followed by biweekly sessions; study by Nasiri et al. (2020) used MBSR for 9 weeks, 2 h per session, once weekly; work by Nyklíček et al. (2014) utilized MBSR, total intervention time lasting for 3 weeks, with weekly 1 session for 90–120 min and, research by Parswani et al. (2013) used MBSR, with a total intervention duration of 8 weeks, weekly 1 session for 60–90 min. Detailed explanation and differences in intervention type, frequency and duration across the selected studies are presented in Table 4.

**Table 3:** Characteristics of reported intervention.

Author, year, and country	Intervention type, duration, and dosage	Mode of delivery	Post-test outcome differences between MBIs and control (Mean ± SD)	Significance level	Effect size (Cohen’s *d*)	Follow-up phase	Authors conclusion
Hou 2019 (China)	MBSR for 6 weeks, three sessions, 30–40 min per session every 2 weeks	Individual, face-to-face followed by two sessions by telephone	Depression and anxiety (HADS)	Depression and anxiety	Depression and Anxiety: 0.58	No follow-up	MBSR program, when compared with routine care showed improved mindfulness and decreased psychological distress.
			MBSR: 10.50 ± 4.37	Significant			
			Control: 14.74 ± 6.90	*p* < 0.001			
			Stress (PSS)	Stress:	Stress: 0.51		
			MBSR: 42.20 ± 8.66	Significant			
			Control: 46.94 ± 8.49	*p* < 0.001			
Jang 2018 (South Korea)	MBAT (MBSR + mindful art therapy) for 12 weeks, 12 sessions, 45 min per session, weekly	Group, face-to-face	Depression (BDI)	Depression:	Depression: 1.58	No follow-up	The MBAT group showed a significant decrease in depression and trait anxiety.
			MBAT: 6.60 ± 3.56	Significant			
			Control: 20.18 ± 11.66	*p* < 0.001			
			Anxiety (TAI)	Anxiety:	Anxiety: 1.86		
			MBAT: 9.56 ± 4.31	Significant			
			Control: 18.58 ± 5.32	*p* < 0.001			
Wu, 2023 (China)	MBSR + Cardiac Rehab for 5–7 days, two sessions/day for 30 min	Individual, face-to-face	Depression (SDS)	Depression:	Depression: 0.134	No follow-up	MBSR intervention combined with early cardiac rehab can alleviate anxiety, depression in AMI patients receiving IABP assistance.
			MBSR: 48.09 ± 2.50	Significant			
			Control: 52.62 ± 4.23	*p* < 0.001			
			Anxiety (SAS)	Anxiety	Anxiety: 0.427		
			MBSR: 53.72 ± 4.40	Significant			
			Control: 58.26 ± 5.52	*p* < 0.001			
Liang 2019 (China)	MBSR for 7 days, seven sessions, 3 h per session, daily	Group of 5–10, face-to-face	Depression (SDS)	Depression:	Depression: 0.46 Anxiety: 0.43	No follow-up	MBSR applied in perioperative period of PCI is beneficial in regulating the negative emotions.
			MBSR: 45.53 ± 6.88	Significant			
			Control: 48.66 ± 6.74	*p* < 0.05			
			Anxiety (SAS)	Anxiety:	Depression: 0.46 Anxiety: 0.43		
			MBSR: 47.18 ± 7.37	Significant			
			Control: 50.41 ± 7.65	*p* < 0.05			
Michalsen 2005 (Germany)	MBSR for1 year, 3 h weekly for first 10 weeks, then 2 h weekly for next 10 weeks and then biweekly	Group of 10–12, face-to-face	Depression (BDI)	Depression, anxiety and stress: Not significant	Depression: 0.29	No follow-up	MBSR program did not improve psychological outcomes in medically stable CAD patients.
			MBSR: 6.4 ± 4.2				
			Control: 9.8 ± 5.8				
			Anxiety (TAI)		Anxiety: 0.03		
			MBSR: 35.7 ± 8.3				
			Control: 37.5 ± 10.0				
			Stress (PSS)		Stress: 0.34		
			MBSR: 19.1 ± 7.6				
			Control: 21.7 ± 7.7				
Nasiri et al. (2020) Iran	MBTP for 9 weeks, 2 h per session, weekly	Not reported	Stress	Significant	Stress: 1.75	No follow-up	It is worthwhile to launch this training program to reduce the stress in patients with acute coronary syndrome.
			MBI: 23.03 ± 7.79				
			Control: 33.75 ± 3.78	*p* < 0.001			
Nyklíček 2014 (Netherlands)	MBSR for 3 weeks, three sessions, 90–120 min per session, weekly	Group	Depression and anxiety (SAD−4)	Anxiety and depression:	Anxiety and depression: 0.92	No follow-up	Mindfulness intervention may be applied to post PCI patients to decrease their levels of psychological symptoms of distress.
			MBSR: 2.42 ± 0.55	Significant *p* < 0.05			
			Control: 2.80 ± 0.54	Stress: Not significant			
			Stress (PSS)		Stress: 0.02		
			MBSR: 18.44 ± 8.23				
			Control: 18.42 ± 6.99				
Parswani 2013 (India)	MBSR for 8 weeks, eight sessions, 1–1.5 h per session, weekly	Individual, face to face	Depression (HADS)	Depression:	Depression:1.05	3 months	MBSR program is effective in reducing symptoms of anxiety and depression and perceived stress in patients with CHD.
			MBSR: 3.33 ± 1.59	Significant			
			Control: 5.47 ± 2.39	*p* < 0.01			
			Anxiety (HADS)	Anxiety:	Anxiety: 1.69		
			MBSR: 3.27 ± 1.27	Significant			
			Control: 7.53 ± 3.33	*p* < 0.001			
			Stress (PSS)	Stress:	Stress: 1.07		
			MBSR: 19.60 ± 3.22	Significant			
			Control: 27.13 ± 9.39	*p* < 0.01			

AMI, Acute myocardial infarction; BDI, Beck’s depression inventory; CAD, Coronary artery disease; CHD, Coronary heart disease; HADS, Hospital anxiety and depression scale; IABP, Intra-aortic balloon pump; MBAT, Mindfulness-based art therapy; MBI, Mindfulness-based intervention; MBSR, Mindfulness-based stress reduction; MBTP, Mindfulness-based training program; PCI, Percutaneous coronary intervention; PSS, Perceived Stress Scale; SAD-4, Modified sad persons scale; SDC, Self-rating depression scale; TAI, Test anxiety inventory.

**Table 4:** Differences in mindfulness-based interventions across selected studies.

Author, year and country	Type and content of intervention	Frequency and duration
Hou 2019 (China)	The standard MBSR, which is a group-based face-to-face program, was modified to one-on-one and face-to-face combined with phone delivery method. In addition, MBSR was adjusted to a simple intervention that included body scan, mindfulness breathing, and sitting meditation. Body scan was conducted in a face-to-face manner during hospital stay while mindfulness breathing and sitting meditation were conducted at home by telephone after discharge from hospital. The standard MBSR is an 8-week, two-and-a-half-hour sessions performed weekly, an all-day retreat, and 45-min mindfulness and yoga exercises practiced at home every day.	This modified MBSR intervention excluded the all-day retreat and mindfulness yoga exercise and shortened the duration of the MBSR from 8- to 6-week and each session lasted for 30–40 min with 20–30 min spent performing the mindfulness intervention and 10 min spent on questions, answers, and scheduling the next intervention.
Jang 2018 (South Korea)	MBAT program based on MBSR was re-structured with a focus on its progress-by-sessions idea. The MBAT group was divided into two sub-groups; A (*n* = 11) and B (*n* = 12), and the same type of treatment was given to both groups. MBAT activities included self-acceptance, awakening the physical sensation, mind is where body is, loving mind, power of breath, realization, from negative to positive, here now and beginners mind.	MBAT was conducted for 12-weeks, each session once weekly lasting 45 min. Focus was emphasized on a single mindfulness art activity that was different during each session.
Wu, 2023 (China)	MBSR was conducted with patients lying on the bed. They were guided to focus on the present moment with openness and acceptance followed by breathing slowly and deeply, placing their hands on their abdomen. The instructor encouraged them to feel their stomach rise and fall, count each breath, and let their heart drift with their breath while remaining focused on the present moment. Finally, they were instructed to concentrate on their quiet breathing and mindfulness rather than any discomfort.	The intervention was performed for 30 min, twice daily and continued until the removal of the IABP, which usually took 5–7 days.
Liang 2019 (China)	MBSR started with patients lying down and focusing on body scanning. Next, they took sitting position and were guided to breath and feel air flowing from nose to abdomen using mindfulness-based breath. This was followed by mindfulness meditation to guide the patients to self-perceive their emotions and thoughts. Afterwards, patients concentrated on walking meditation to feel the contact between the foot and the ground. Finally, mindfulness-based introspection was implemented to control emotions and correctly deal with negative emotions.	MBSR begun 3–5 days after PCI and intervention was run for 7 consecutive days, once daily for 1 h. Each of the five steps mentioned in the intervention type were repeated 3–4 times, 5 min each time.
Michalsen 2005 (Germany)	Patients practiced the stress reduction technique based on their personal preferences, which included mindfulness meditation, guided imagery, yoga breathing, and body scan. The program also incorporated coping skills training. Patients learned that emotions and behaviors are largely influenced by their perceptions and thoughts. Additionally, the program featured practical exercises to foster non-judgment, acceptance, and patience.	The 1-year intervention was carried out in small groups of 10–12 participants in order to ensure group support. The program started with a 3-day retreat followed by weekly 3-h sessions for 10 weeks and thereafter by biweekly 2-h sessions.
Nasiri et al. (2020) Iran	MBTP incorporated different mindfulness activates every week such as recognition of excitement, beauty of nature, focus on single object and single movement, inner and outer experience, concept of physical mindfulness, awareness of feeling and excitement, listening to unpleasant excitement, recognition of thoughts, concept of thought in mindfulness, understanding the concepts of body and feeling mindfulness, mindful breathing, and mindfulness in mindful activities.	Intervention lasted 9-week, with one session every week for 2 h. The emphasis was placed on unique mindfulness activities that varied with each session.
Nyklíček 2014 (Netherlands)	The modified MBSR program consisted of four components that included psychoeducation on how behavior, bodily sensations, emotions, and thoughts contribute to psychological distress; psychoeducation on how mindfulness and nonjudgmental acceptance of bodily sensations, thoughts, feelings, and behavior can reduce stress; mindfulness practices focusing on bodily sensations, emotions, and thoughts while sitting upright and discussion of personal experiences with these practices both during sessions and at home.	The revised program in this study was less intensive, consisting of just three weekly sessions of 90–120 min each, plus an additional evaluation session 2 weeks later.
Parswani 2013 (India)	The intervention program consisted of training in various forms of mindfulness meditation, including body scan meditation, sitting meditation, mindful walking, mindful eating, the 3-min breathing space, mastery and pleasure activities, and cognitive restructuring. Each participant received an audio cassette with recorded instructions for mindfulness and body scan meditation to facilitate 30 min of home practice.	This was an 8-week MBSR intervention and sessions were held once a week, with each session lasting for 1–1.5 h.

IABP, Intra-aortic balloon pump; MBAT, Mindfulness-based art therapy; MBSR, Mindfulness-based stress reduction; MBTP, Mindfulness-based training program; PCI, Percutaneous coronary intervention.

### 3.3 Comparator characteristics

By interpreting Table 2, two kinds of control methods were reported in the articles analyzed, inactive and active control methods. Active control is where the control group receives a different intervention contemporaneously with the experimental group, whereas inactive control does not receive any comparison treatment throughout the study or receives treatment at the end of study (Byrd-Bredbenner et al., 2017). Trials by Hou et al. (2019) and Jang et al. (2018) used inactive control group of waitlist control; study by Liang et al. (2019) utilized inactive control group of conventional nursing care; research by Wu et al. (2023) had active control group who participated in early cardiac rehabilitation; experiment by Michalsen et al. (2005) employed active control group of written advice only; study by Nasiri et al. (2020) had inactive control group of routine care; and trial by Nyklíček et al. (2014) used active control group of self-help booklet containing all the exercises, while study by Parswani et al. (2013) had inactive control group of routine cardiac care.

### 3.4 Psychometric measures of mental health

Participants’ mental health was evaluated with different psychometric scales. Seven trials examined the anxiety and depression symptoms, whereas five studies investigated stress symptoms in addition to other two outcomes. Depression symptoms were assessed utilizing the Beck Depression Inventory—BDI (Michalsen et al., 2005; Jang et al., 2018), the depression subscale of the Hospital Anxiety and Depression Scale—HADS (Parswani et al., 2013; Hou et al., 2019), the Self-rating Depression Scale—SDS (Liang et al., 2019; Wu et al., 2023), and the Symptoms of Anxiety-Depression index—SAD (Nyklíček et al., 2014). The anxiety symptoms were examined using the anxiety subscale of HADS (Parswani et al., 2013; Hou et al., 2019), the Self-rating Anxiety Scale—SAS (Liang et al., 2019; Wu et al., 2023), the Trait Anxiety Inventory—TAI (Jang et al., 2018), the State and Trait Anxiety Inventory—STAI (Michalsen et al., 2005), and SAD (Nyklíček et al., 2014). Five studies gauged stress symptoms using the Perceived Stress Scale—PSS (Michalsen et al., 2005; Parswani et al., 2013; Nyklíček et al., 2014; Hou et al., 2019; Nasiri et al., 2020).

### 3.5 Risk of bias assessment

**Figure 2:**
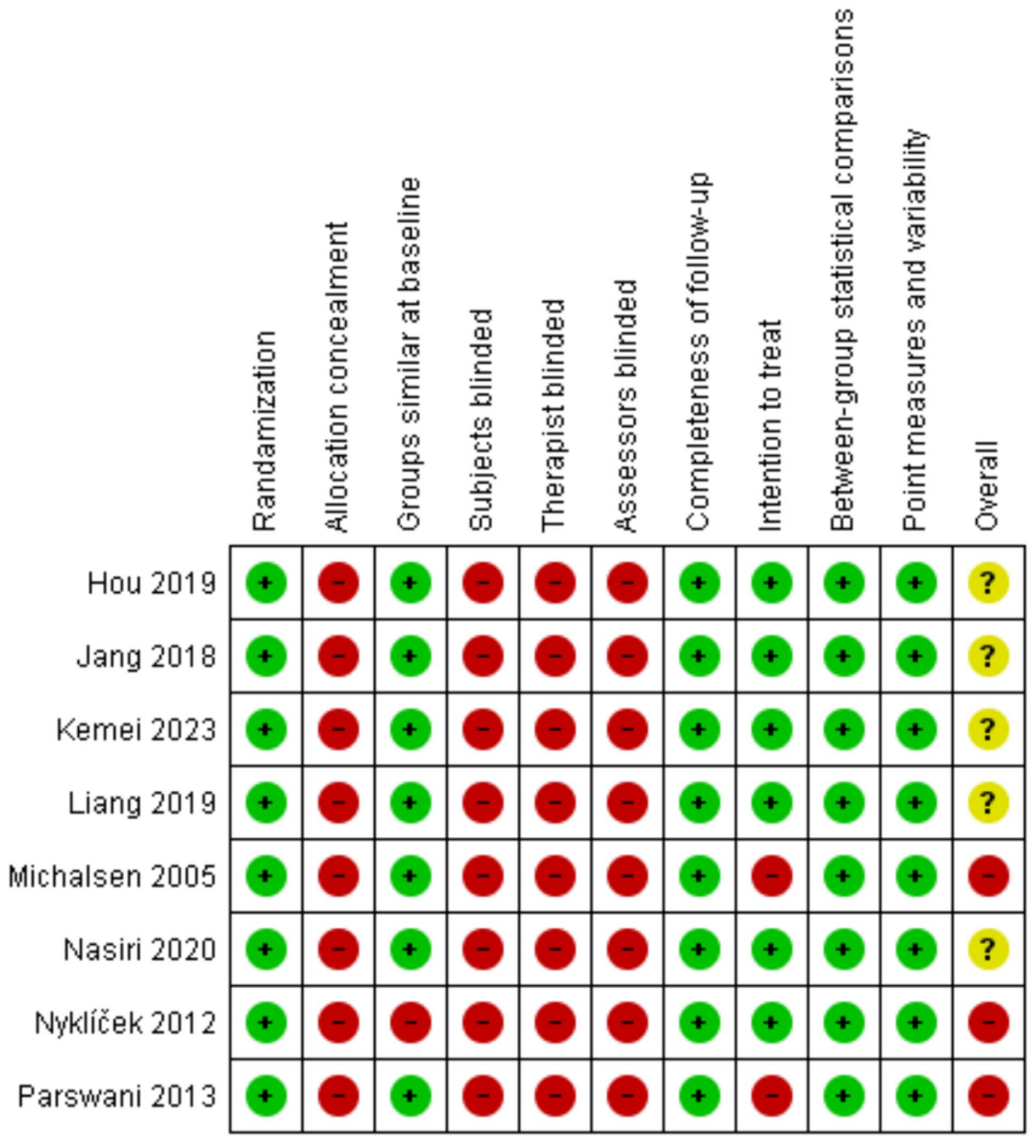
Risk of bias—PEDro score.

Risk of bias and completeness of statistical reporting of RCTs were assessed using the PEDro scale. PEDro scale has good construct validity and fair to good reliability (Maher et al., 2003; de Morton, 2009). The scale contains 11 questions (eligibility criteria, randomization, allocation concealment, similarity at baseline, subject-blinded, therapist-blinded, assessor-blinded, completeness of follow-up, intention to treat, between-group statistical comparisons, and point measures, and variability) which are used to determine the level of quality regarding the studies’ research methods. Apart from question number one, each question that gets a “yes” will increase the assessment score by 1. With the range of score from 0 to 10, studies with scores of 0–3 are considered “poor,” 4–5 “fair,” 6–8 “good,” and 9–10 “excellent.” This assessment scale was not used as a determinant on whether a study should be included but served as the interpreter on the level of evidence-based practice in studies that have been included. All articles included in this review achieved the criteria of randomization and baseline comparability of participants in intervention and control groups. None of the study achieved the allocation concealment and no study blinded the subjects, therapists and/or outcome accessors. All studies obtained the key outcomes on follow-up and five trials achieved the intention to treat criteria. All trials carried out between group statistical analysis, and point measures and variability as shown in Figure 2. Five studies obtained a “good” PEDro score with six points (Jang et al., 2018; Hou et al., 2019; Liang et al., 2019; Nasiri et al., 2020; Wu et al., 2023), and three articles obtained “fair” with five points (Michalsen et al., 2005; Parswani et al., 2013; Nyklíček et al., 2014). In general, methodological quality of five studies (62.5%) met with some concerns while three trials (37.5%) had high risk of bias.

### 3.6 Effect of MBIs on mental health

**Figure 3:**
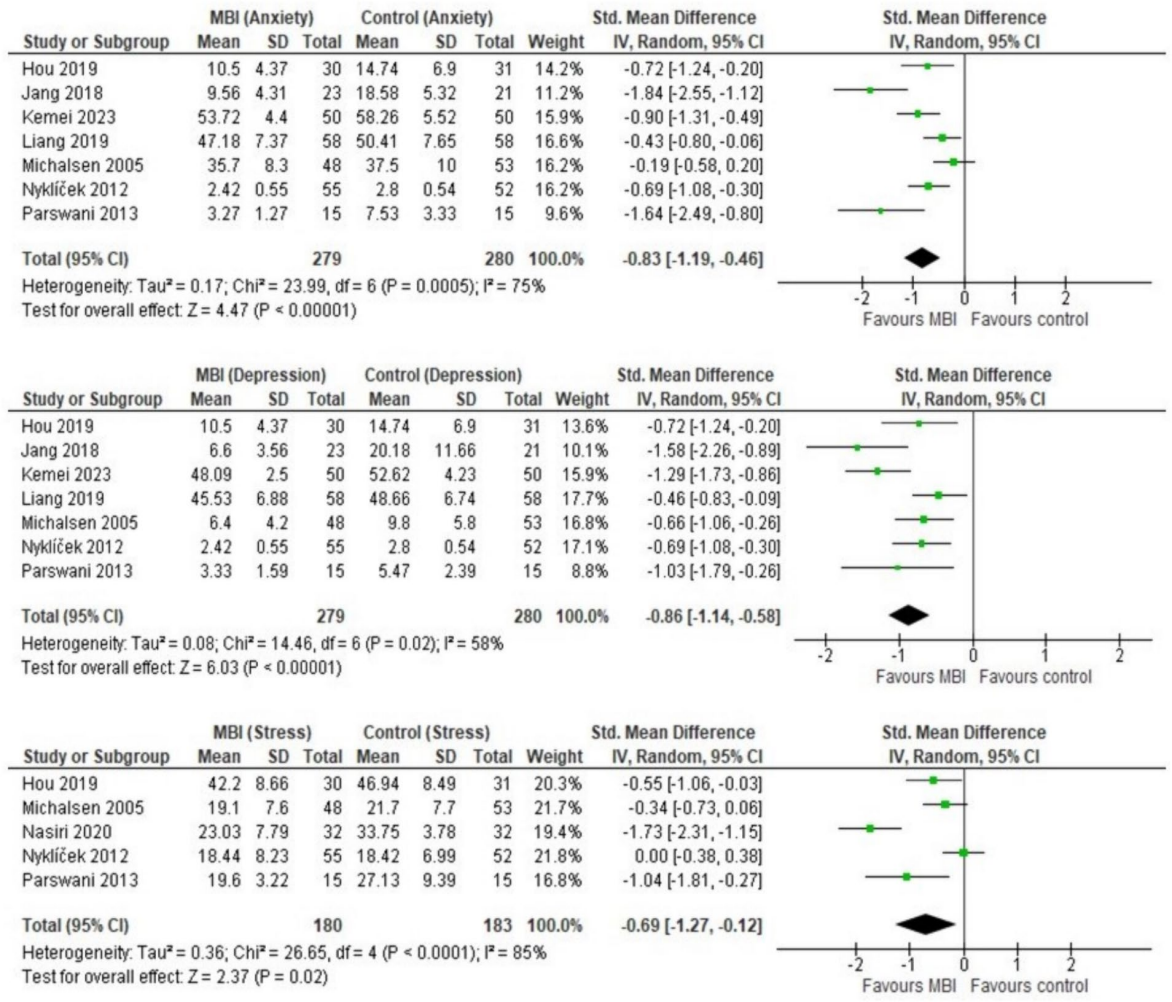
Forest plot: MBI vs. control on anxiety, depression, and stress.

**Figure 4:**
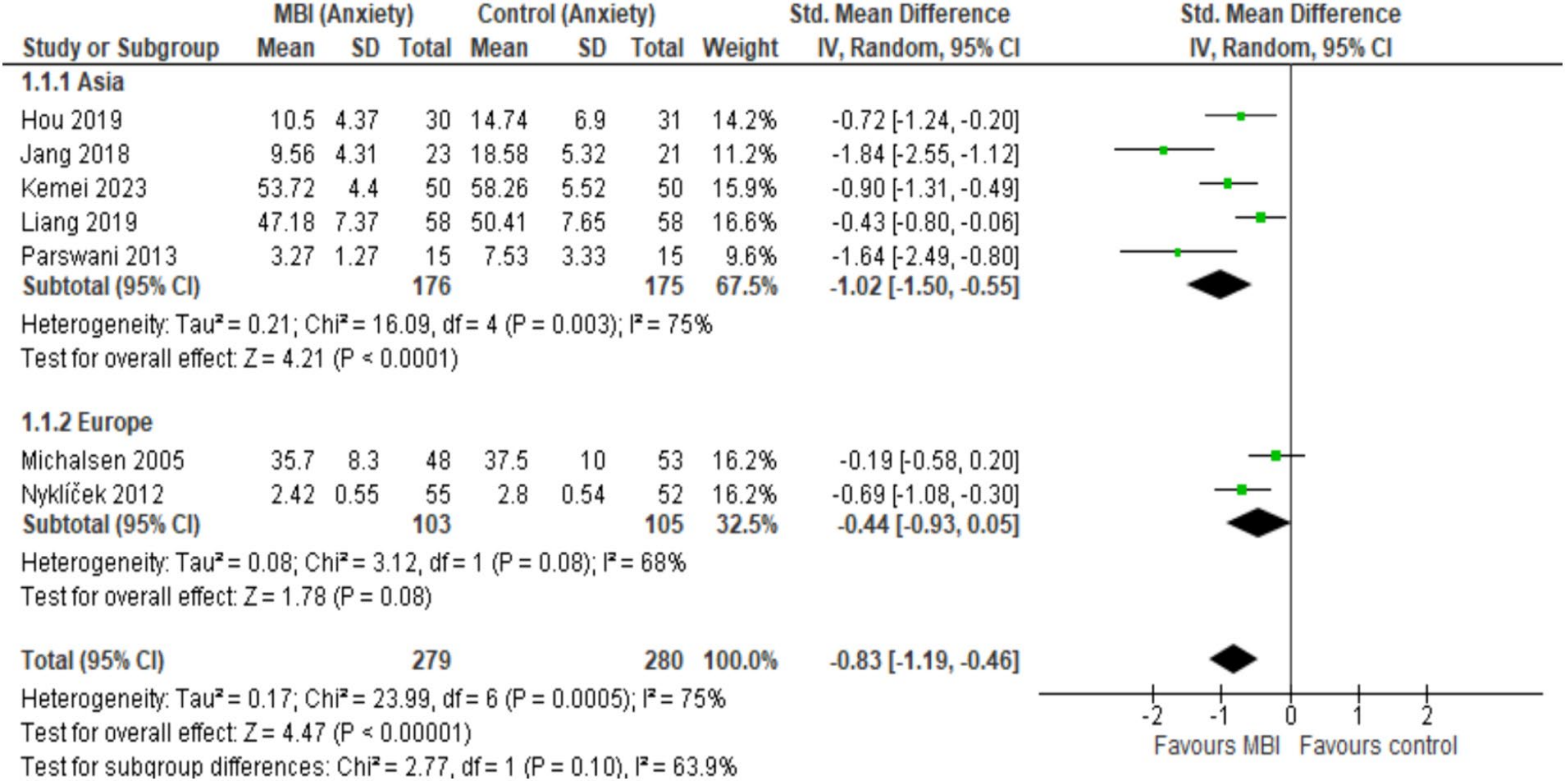
Forest plot comparison of anxiety: Asia vs. Europe.

**Figure 5:**
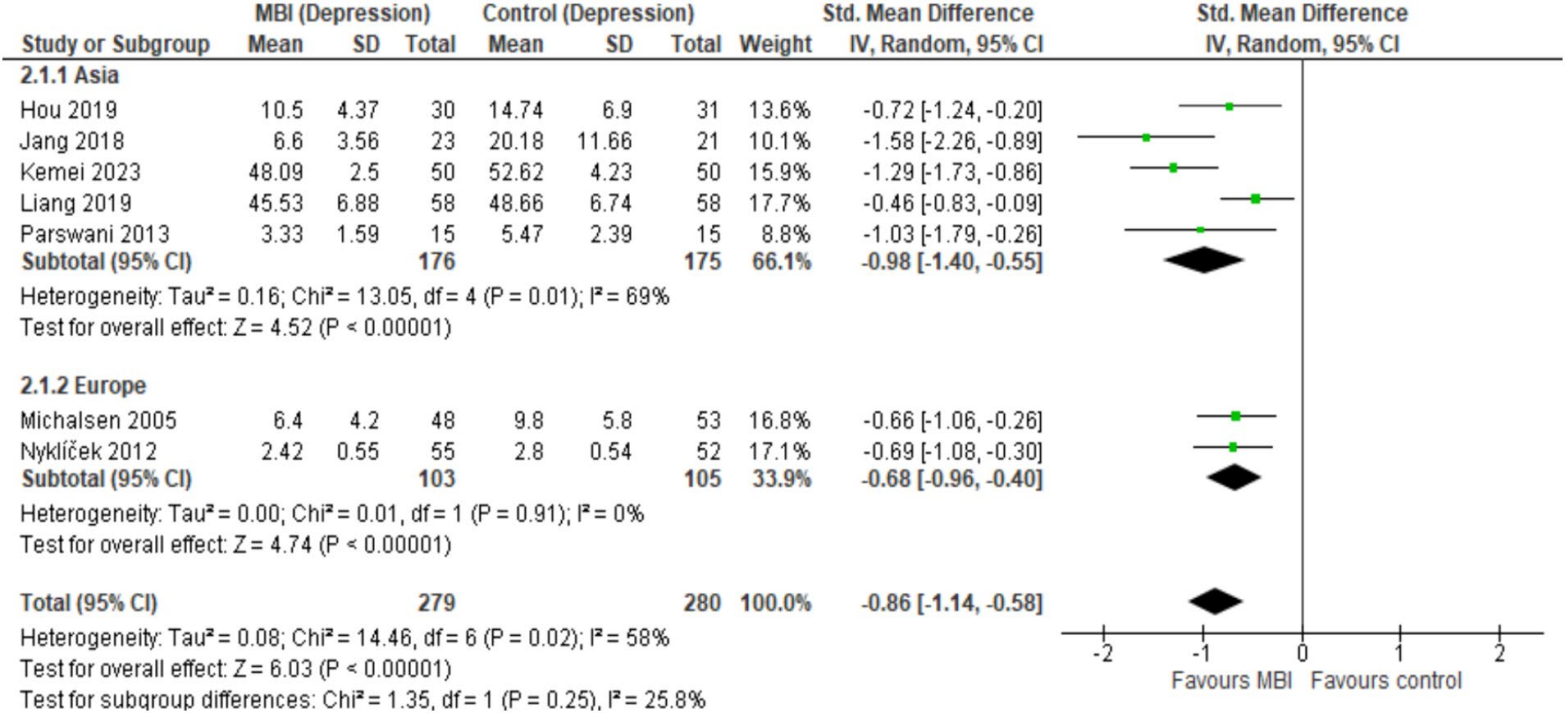
Forest plot comparison of depression: Asia vs. Europe.

**Figure 6:**
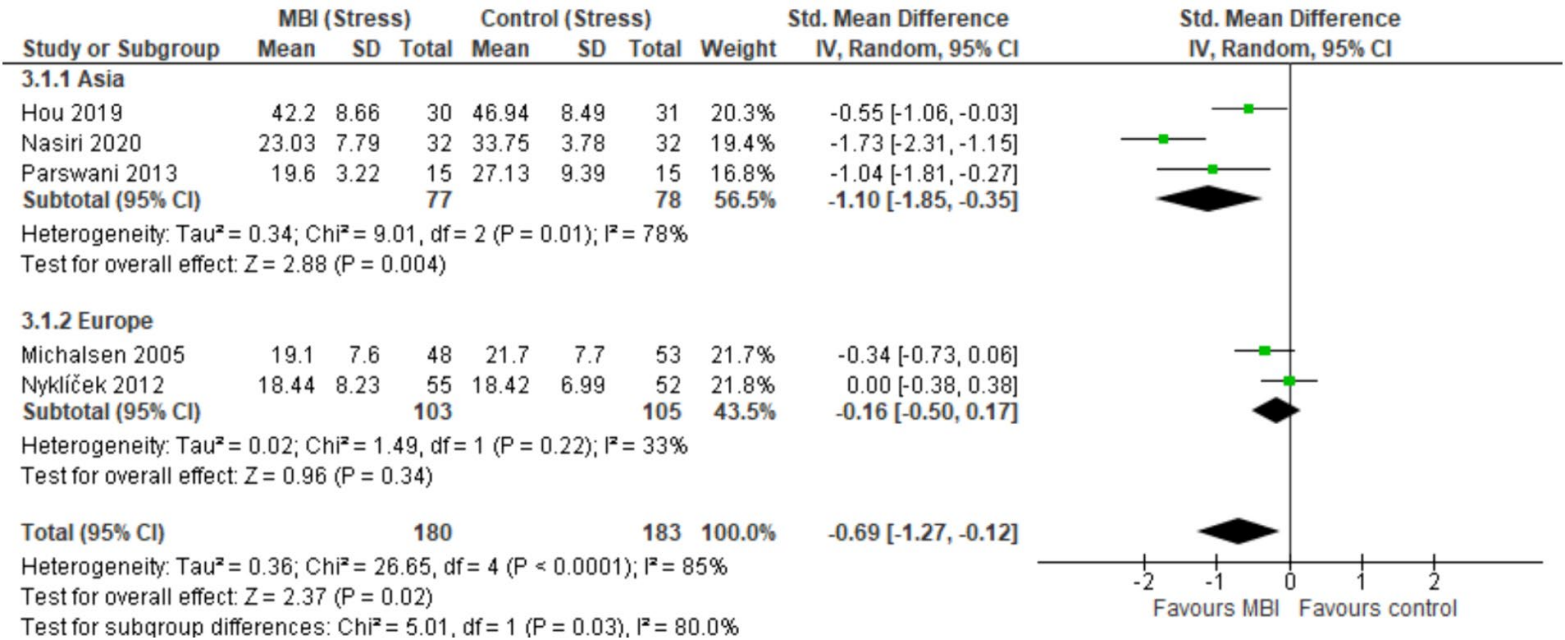
Forest plot comparison of stress: Asia vs. Europe.

Overall, CAD patients in the intervention arms showed statistically significant and beneficial effects in the mental health outcomes as a result of MBI participation (Figure 3). The pooled results from seven studies (*n* = 559) showed a statistically significant effect of MBIs on anxiety (SMD = −0.83; 95% CI [−1.19, −0.46], *p* < 0.001, *I*^2^ = 75%) and depression (SMD = −0.86; 95%CI [−1.14, −0.58], *p* < 0.001, *I*^2^ = 58%). A statistically significant effect was reported across five studies (*n* = 363), that investigated the effect of BMIs on stress (SMD = −0.69; 95% CI [−1.27, −0.12], *p* = 0.02, *I*^2^ = 85%). Sensitivity analyses revealed that regionally (Asia vs. Europe), there were no statistically significant differences in MBI outcomes for anxiety (*I*^2^ = 63.9%, *p* = 0.10) and depression (*I*^2^ = 25.8%, *p* = 0.25), suggesting that intervention is not influenced based on the region (Figures 4, 5). Figure 6 depicts the effect of MBIs on stress with a significant difference (*I*^2^ = 80.0%, *p* = 0.03) between Asian and European subgroups. However, a small number of studies with fewer participants contributed to the data of European subgroup (two studies, 208 participants) compared to Asian subgroup (five trials, 351 participants) for anxiety and depression, whereas for the stress, there were three trials in Asian subgroup (155 participants) in comparison to two studies in European subgroup (208 participants) which indicates an uneven covariate distribution and this subgroup analysis is unlikely to produce any useful findings. In addition, there is substantial amount of heterogeneity in each subgroup for anxiety (Asia: *I*^2^ = 75%, Europe: *I*^2^ = 68%), a moderate amount of heterogeneity for depression in Asian subgroup (*I*^2^ = 69%) and substantial amount of heterogeneity within each subgroup for stress (Asia: *I*^2^ = 78%, Europe: *I*^2^ = 33%) which could be attributed to uneven covariate distribution.

## 4 Discussion

The current systematic review and meta-analysis compiled evidence from eight RCTs to highlight the efficacy of MBIs on the mental health of patients with CAD. Since past literature (Scott-Sheldon et al., 2020) primarily targeted CVD patients, the authors of the current study specifically focused on CAD population and quantified the effectiveness of MBIs on three crucial psychological domains. It was found that MBIs were more likely to alleviate mental health issues concerning symptoms of anxiety, depression, and stress in experimental groups compared to control groups. The most notable effect sizes were evident for symptoms of depression (*Z* = 6.03), followed by anxiety (*Z* = 4.47) and stress (*Z* = 2.08). The findings of this analysis were in line with past systemic reviews of MBIs conducted among different target populations such as patients with vascular diseases (Abbott et al., 2014), CVDs (Scott-Sheldon et al., 2020), and heart diseases (Zuraida and Syahrul, 2019). All these studies concluded the effectiveness of MBIs in providing psychological and physiological benefits to the patients. Our qualitative findings indicated that the majority of studies demonstrated significant differences between intervention and control groups except one study which reported non-significant changes in depression and anxiety (Michalsen et al., 2005), and two studies reported non-significant changes in stress (Michalsen et al., 2005; Nyklíček et al., 2014). The type, frequency, and duration of mindfulness intervention used in each trial could be a factor in determining the effectiveness of the intervention, as it may produce different effects. Therefore, an inference can be drawn that due to the different intervention protocols used in each study, there were some possible differences in the psychological outcomes between control and intervention groups. However, when the results were corroborated by an extensive meta-analysis, pooled effect sizes indicated notable psychological improvements in patients employing MBIs. We hypothesize these positive psychological and mental health outcomes occur as a result of changes in brain thickness and functional network connectivity (Chiesa and Serretti, 2010; Yang et al., 2016). Studies investigating the effect of BMIs reported that brain regions and subcortical areas related to attentional processes are thicker in those practicing meditation compared to controls, where sustained voluntary attention and regulation decreases the exaggerated emotional reactivity and provide a basis for balanced mind–body interaction associated with positive psychological outcomes (Chiesa and Serretti, 2010). In addition, a functional magnetic resonance imaging study found significant differences in the functional connectivity of different brain regions (precuneus, temporoparietal junction, dorsal anterior cingulate cortex, anterior insula, pregenual anterior cingulate, and dorsomedial prefrontal cortex) before and after the mindfulness intervention, suggesting its therapeutic usefulness by producing plasticity related brain network changes and altering the neuronal basis of anxiety and depression (Yang et al., 2016). The therapeutic effects of MBIs function on various constructs such as mindfulness, rumination, worry, cognitive and emotional reactivity, self-compassion, and psychological flexibility (Gu et al., 2015; Shapero et al., 2018). This aligns with the theoretical underpinnings of MBSR, which supports the non-reactivity or acceptance mechanism (Kabat-Zinn, 1982) and increasing positive psychological attributes (Shapero et al., 2018). Evidence for the mechanisms of mindfulness and emotional reactivity supports the central theories of MBSR, which state that developing mindfulness skills can foster insight and non-reactive acceptance of one’s experience for positive beneficial outcomes. A past review (Hofmann and Gómez, 2017) discovered that MBIs are superior to waitlists, psychoeducation, supportive psychotherapy, relaxation training, visualization, or suppression strategies due to their ability to lessen the severity of psychological and physiological symptoms concurrently. Hence, MBIs have been proven to be an effective alternative therapy in lowering the intensity of anxiety and depressive symptoms in a variety of chronically ill patients.

There were a few shortcomings in the study by Michalsen et al. (2005) which might be accountable for the lack of psychological improvement when compared to active control vs. MBSR and are worth mentioning. Firstly, it should be noted that the symptoms of anxiety and depression often disappear on their own within a few weeks or a few months after an episode of acute coronary syndrome or after undergoing a revascularization surgery (Mathisen et al., 2007; Barnason et al., 2012; Kim et al., 2022). The participants in Michalsen et al. (2005) were stable and had recently undergone their treatment with at least 3 months of no acute coronary syndrome. Furthermore, out of 105 participants, only seven were re-hospitalized during the subsequent 1 year of treatment period as majority of them were given elective hospitalization options. It can be suspected that staying at home during the study period could have produced a positive impact on their mental health regardless of whether they were exposed to the intervention or not. According to Michalsen et al. (2005), improving level of mental state is normal in the cycle of CAD. Mental health normally would take around 2 weeks to a month to improve, assuming there are no secondary complications. This would allow the patient to recuperate with their family members and in the comfort of their home, leading to a positive environmental factor that further boosts their mental health. Secondly, their study also yielded a significant reduction in depression only among females. It might be due to the gender-specific benefits provided by MBSR. From the data obtained, it is shown that women diagnosed with CAD tend to be older and more likely to have secondary complications compared to men. Since females tend to have higher vulnerability to psychological issues, they may show greater sensitivity toward the elements used in the MBSR program, which eventually leads to a significant impact of the intervention on female subjects. Hence, it can be inferred that gender-specific outcomes are also an important factor to determine the efficacy of MBIs in CAD patients. A study (Emery et al., 2004) stated that females diagnosed with cardiac conditions tend to have greater mental distress in the form of anxiety when compared to the opposite gender. This finding further solidifies that the efficacy of MBIs may differ across both genders and may be accountable for the difference in results. Whereas, the other study by Nyklíček et al. (2014) which reported non-significant findings could not satisfy the baseline comparability of the PEDro scale, which places the article to have a potential risk of bias.

Several limitations were taken into careful consideration by the researchers. First, the small sample size across the studies led to a lesser number of participants in follow-up, which further reduced the validity of the outcome measures. There was also a disproportionate ratio of male and female participants. Studies that only included male participants, could limit the generalizability of the outcome measure due to representation bias. Some studies that implemented the MBSR program had shortened intervention time frame and follow-up duration which may have attenuated the intervention effects. Next, some of the studies reported to have spent more time and attention toward the intervention group than the control group, this could significantly increase the risk of bias toward the intervention group. This further led to a higher drop-out rate of participants in the control group, due to the lack of motivation and drive to continue their treatment. There were also notable inadequacies in the types of control groups used as comparators. Studies should incorporate both kinds of control groups to determine the effectiveness of an experimental intervention in relevance to an established treatment. Lastly, since English-based databases were only consulted, there is a higher possibility that studies reported in languages other than English or published in non-mainstream databases might not have been included; consequently, leading to the potential risk of publication bias.

According to current literature, men typically have shown an increased CAD prevalence with earlier onset, higher behavioral risk factors, and obstructive heart conditions (Lima Dos Santos et al., 2023). The predominance of male subjects found in this study provides valuable insights, however, their generalizability, especially in gender-specific health outcomes involving CAD, can be limited. Therefore, addressing these limitations involves careful consideration of gender-specific factors (e.g., biological, hormonal, psychological, behavioral, etc.) in controlling the potential impact of confounders and ensuring adequate representation of both genders to enhance the validity and applicability of research findings across diverse populations. In addition, potential recruitment strategies involving multicenter trials, population-based registries, and international research collaborations should be adopted by future researchers to address the limitations posed by small sample sizes and ensure adequate sample sizes. The duration and dosage of mindfulness interventions play pivotal roles in determining its effectiveness, feasibility, and sustainability; thus, it should be also taken into careful consideration. Longer duration of MBIs may allow participants more time to internalize mindfulness practices; whereas, higher dosage frequency may yield an increased therapeutic effect. Nevertheless, longer interventions or higher dosages may lead to a higher dropout among CAD patients due to health-related constraints or personal factors. Therefore, tailoring intervention durations and dosages to the specific needs and capabilities of CAD patients is crucial for maximizing adherence and minimizing attrition. MBIs have proven to demonstrate short-term psychological benefits, but their long-term sustainability requires further investigation and validation. CAD patients often experience chronic stress, anxiety, and depression, which can worsen their cardiovascular health outcomes and overall quality of life over time. Hence, the short-term benefits observed immediately after MBIs may not reflect the long-term psychological stability or improvement. Assessing sustainability through long-term follow-up can help determine the durability of psychological benefits beyond the MBI intervention period, and ensure the effective use of mindfulness practices in patient’s daily life, even after the initial reductions in anxiety, depression, and stress levels.

## 5 Conclusion

This review concludes that MBIs have a higher efficacy in improving the psychological outcomes of anxiety and depression among patients with CAD compared to subjects in the inactive control arm. The effectiveness of MBIs on stress is mostly positive but dependent on the type of mindfulness-based program and type of control group used. Although MBIs demonstrate promising short-term benefits for psychological well-being, understanding the sustainability of these effects requires rigorous long-term follow-up studies. Therefore, it is imperative to conduct more rigorous and robust studies with a larger sample, equal gender ratio and long-term follow-up to accurately measure the effectiveness of MBIs. Future research efforts should prioritize longitudinal designs, incorporate diverse methodologies, and investigate factors influencing sustained mindfulness practice and psychological benefits. Simultaneously, integrating the key implications of MBIs in clinical practices through effective patient education, tailored interventions, and patient-centered care plans may help to enhance the effectiveness of MBIs in promoting sustained mental health outcomes and improving the CAD patient outcomes and quality of life over time. It is also necessary to explore the potential factors that may contribute to long-term mindfulness practice and greater psychological well-being. Considering a holistic biopsychosocial approach and including personal motivation, ongoing support system, integration of mindfulness into daily routines, and balancing life stressors may optimize the use of MBIs to foster enduring improvements in psychological well-being and enhancing overall mental health outcomes. By addressing these crucial aspects, future researchers can better facilitate clinical practitioners and policy-decision makers regarding the integration of MBIs in mental health care for long-term benefits.

## Data availability statement

The original contributions presented in the study are included in the article/supplementary material; further inquiries can be directed to the corresponding author.

## Author contributions

HA: Formal analysis, Writing – review & editing, Investigation. IM: Formal analysis, Writing – review & editing, Conceptualization, Funding acquisition, Methodology, Writing – original draft. SH: Formal analysis, Writing – original draft, Writing – review & editing. RT: Data curation, Investigation, Writing – original draft. DV: Writing – review & editing, Data curation.
